# Feruloyl Esterase (*La*Fae) from *Lactobacillus acidophilus*: Structural Insights and Functional Characterization for Application in Ferulic Acid Production

**DOI:** 10.3390/ijms241311170

**Published:** 2023-07-06

**Authors:** Sangeun Jeon, Jisub Hwang, Hackwon Do, Ly Thi Huong Luu Le, Chang Woo Lee, Wanki Yoo, Min Ju Lee, Seung Chul Shin, Kyeong Kyu Kim, Han-Woo Kim, Jun Hyuck Lee

**Affiliations:** 1 Department of Chemistry, College of Natural Science, Sookmyung Women’s University, Seoul 04310, Republic of Korea; 2 Research Unit of Cryogenic Novel Material, Korea Polar Research Institute, Incheon 07505, Republic of Korea; 3 Department of Polar Sciences, University of Science and Technology, Incheon 07505, Republic of Korea; 4 Department of Precision Medicine, Graduate School of Basic Medical Science (GSBMS), Sungkyunkwan University School of Medicine, Suwon 16419, Republic of Korea; 5 Division of Life Sciences, Korea Polar Research Institute, Incheon 07505, Republic of Korea; ssc@kopri.re.kr

**Keywords:** crystal structure, *La*Fae, ferulic acid, feruloyl esterase, X-ray crystallography

## Abstract

Ferulic acid and related hydroxycinnamic acids, used as antioxidants and preservatives in the food, cosmetic, pharmaceutical and biotechnology industries, are among the most abundant phenolic compounds present in plant biomass. Identification of novel compounds that can produce ferulic acid and hydroxycinnamic acids, that are safe and can be mass-produced, is critical for the sustainability of these industries. In this study, we aimed to obtain and characterize a feruloyl esterase (*La*Fae) from *Lactobacillus acidophilus*. Our results demonstrated that *La*Fae reacts with ethyl ferulate and can be used to effectively produce ferulic acid from wheat bran, rice bran and corn stalks. In addition, xylanase supplementation was found to enhance *La*Fae enzymatic hydrolysis, thereby augmenting ferulic acid production. To further investigate the active site configuration of *La*Fae, crystal structures of unliganded and ethyl ferulate-bound *La*Fae were determined at 2.3 and 2.19 Å resolutions, respectively. Structural analysis shows that a Phe34 residue, located at the active site entrance, acts as a gatekeeper residue and controls substrate binding. Mutating this Phe34 to Ala produced an approximately 1.6-fold increase in *La*Fae activity against *p*-nitrophenyl butyrate. Our results highlight the considerable application potential of *La*Fae to produce ferulic acid from plant biomass and agricultural by-products.

## 1. Introduction

Hydroxycinnamic acids (HCAs), such as ferulic and caffeic acids, are one of the most widely distributed naturally occurring phenolic acids, typically present in the form of esters and found mainly in grains, fruits and vegetables. They function in crosslinking lignin and hemicellulose of the plant cell wall, thereby noticeably enhancing their physical strength and mechanical properties [1,2]. Recently there has been a growing interest in industrial usages of HCAs, as these compounds have been shown to exhibit anti-inflammatory, antioxidant, neuroprotective, anti-aging, anti-carcinogenic and antimicrobial properties [3,4].

Feruloyl esterases (FAEs, E.C. 3.1.1.73), a subfamily of carboxylic acid esterases, are mainly associated with the release and exploitation of HCAs from plant cell walls [5,6]. These enzymes can further facilitate the binding of other enzymes and generate lignin-carbohydrate complexes for subsequent biodegradation. FAEs are classified into four subgroups (denoted A, B, C and D) based on their primary sequences and catalytic properties [7,8]. Furthermore, these esterases interact with other cellulases or xylanases, thereby enhancing the biodegradation of wheat bran, corncobs, corn bran and grass lignocellulose materials [9,10]. Accordingly, FAEs appear to have considerable potential with respect to numerous industrial and food-related applications, such as animal feed supplements, biofuel production and bioactive component synthesis. Thus, the discovery of FAEs with improved properties using protein-engineering methods is a noteworthy pursuit. For example, mutants designed and generated based on the simulated structure of FAE from *Lactobacillus acidophilus* showed an increased ferulic acid production from agricultural waste [11]. 

Although several fungal FAEs have been characterized, there is currently a growing interest in the potential utility of FAEs derived from lactic acid bacteria (LAB), due to their safety and health-promoting properties. To date, a range of LAB species, including *Bifidobacterium animalis* [12], *Lactobacillus helveticus* [13], and *Lactobacillus plantarum* [14], have been shown to produce FAEs, although only limited information is available regarding their functional properties, structural characteristics and bioavailability. Given that LAB strains are fermentative, easy to culture and safe to use, from a practical perspective, we considered that it would be prudent to investigate FAEs produced by *L. acidophilus*, which is among the most widely-known probiotic bacteria associated with a diverse range of foods, including dairy products, vegetables, meat and fish [15,16]. In addition, *L. acidophilus* is one of the most useful bacterial species, particularly with respect to the biotechnological preparations of food products and dairy supplements [17,18]. Given the industrial significance of FAEs and their roles in LAB, in the present study, we sought to identify and characterize a feruloyl esterase (*La*Fae) from *L. acidophilus*.

Following the identification of a candidate esterase, we evaluated its biotechnological potential based on its capacity to release ferulic acid from wheat bran, rice bran and corn stalks (CSs) when applied in conjunction with xylanase.

## 2. Results and Discussion

### 2.1. Bioinformatic Analysis of LaFae

Following bioinformatic analyses, a gene encoding an FAE (*La*Fae, GeneBank I.D.: EEJ76955.1) was identified in *L. acidophilus* [19]. The phylogenetic relationship between *La*Fae and other FAEs, including FaeLam from *Lactobacillus amylovorus*, was examined and is shown in a phylogenetic tree (Supplementary Figure S1A,B) [20]. Most FAEs from LAB are classified as type C. *La*Fae and its homologs are found not only in *L. acidophilus*, but have also been identified in other *Lactobacillus* species, thereby indicating its widespread distribution in this genus. It is also worth noting that gene cluster analyses indicated that genes encoding phosphoglycerate mutase and *La*Fae are adjacently located within the genome of *Lactobacillus* species (Supplementary Figure S1C). Considering that *La*Fae has a sequence similarity of 83.4% identity and 90.89% similarity with *L. amylovorus* (AOR52353) and shows clustering patterns with other known FAEs, we annotate *La*Fae as a feruloyl esterase.

### 2.2. Characterizations of LaFae

**Figure 1:**
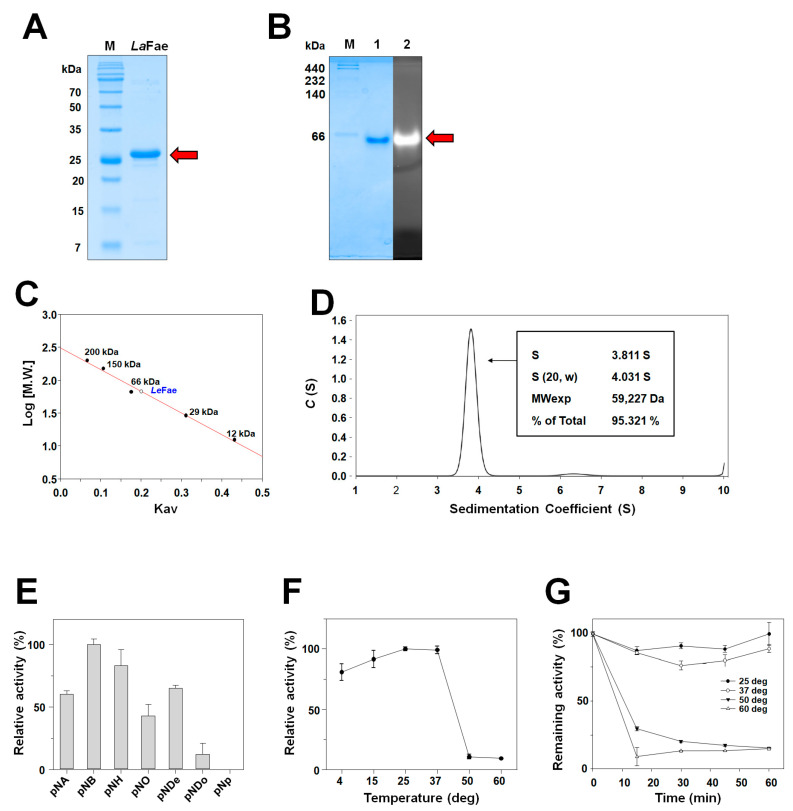
Biochemical characterization of *La*Fae. (**A**) SDS-PAGE analysis of *La*Fae showing the size of monomeric *La*Fae. (**B**) Zymographic analysis of *La*Fae showed the size of *La*Fae through Native-PAGE. (**C**) Gel-filtration chromatographic analysis of *La*Fae shows that it exists as a dimer in solution. (**D**) The result of analytical ultracentrifugation (AUC) indicated that *La*Fae is a stable dimer in solution. (**E**) Substrate specificity analysis of *La*Fae using different chain lengths of *p*-nitrophenyl esters. (**F**) Optimal temperature for *La*Fae activity. The activity value obtained using *p*NB as a substrate at 37 °C was considered 100%. (**G**) Thermal stability of *La*Fae. Activity at 0 min was considered as 100% for all four temperatures at which the stability was studied. *La*Fae, *L. acidophilus* feruloyl esterase; *p*NB, *p*-nitrophenyl butyrate.

In the present study, we used immobilized Ni^2+^-affinity chromatography to purify *La*Fae as a His_6_-tagged fusion protein. As shown in Figure 1A, *La*Fae has a molecular weight of 29 kDa in SDS-PAGE. In addition, strong fluorescence based on activity staining in native-PAGE was detected (Figure 1B). Furthermore, gel-filtration chromatography indicated that *La*Fae tends to polymerize into a dimeric conformation (Figure 1C); the *La*Fae dimerization in solution was confirmed by the analytical ultracentrifugation (AUC) experimental results. A major peak (95.3%) of dimer appeared at 3.811 sedimentation coefficient position, corresponding to 59.2 kDa (Figure 1D). The calculated molecular weight of the *La*Fae monomer was 27.37 kDa based on the amino acid sequence (Figure 1D). As shown in Figure 1E, *La*Fae appears to have a strong preference for short or medium-length substrates, including *p*-nitrophenyl butyrate (*p*NB), *p*-nitrophenyl hexanoate (*p*NH) and *p*-nitrophenyl decanoate (*p*NDe). Similar behavior has been observed in other FAEs, including FaeI from *Cellulosilyticum ruminicola* [21] and Lp_0796 from *L. plantarum* [14]. We discovered that the activity of *La*Fae is optimal within a temperature range of 25 and 37 °C and shows approximately 75% of the optimal activity at 4 °C (Figure 1F). The *La*Fae thermostability was determined by incubation for 1 h at temperatures ranging between 25 and 60 °C, which revealed that the activity of *La*Fae was not substantially altered at temperatures 25 or 37 °C, whereas 70% of the activity disappeared after a 15 min incubation at 50 °C (Figure 1G).

The optimum pH for *La*Fae activity was found to be 8.0, whereas it retained, approximately, 70% activity at pH 7.0 (Supplementary Figure S2A). Furthermore, we discovered that *La*Fae retained approximately 65 and 20% of its activity in the presence of 10 and 30% ethanol, respectively, whereas approximately 35% of activity was preserved in the presence of 0.1% SDS (Supplementary Figure S2B). However, only 24% of activity was detected when the enzyme was exposed to 1% of both, Triton X-100 and Tween 20. Interestingly, we observed that the activity of *La*Fae increased with the increase in NaCl concentration. Specifically, relative activities of 260 and 280% were detected at 1.0 M and 2.0 M NaCl, respectively. Alternatively, only small changes in activity (0–20%) were detected with increasing glycerol concentration (Supplementary Figure S2C).

In addition, we noted a gradual reduction of enzyme activity with the increase in urea concentration. *La*Fae retained 68% of its original activity when exposed to 0.5 M urea and approximately 25% of its activity remained when the concentration of urea exceeded 2.0 M (Supplementary Figure S2D). Far-UV CD spectra in range 210–220 nm in the presence of urea supported the finding that the secondary structure of *La*Fae was not completely destroyed in 2.0 M of urea solution (Supplementary Figure S2E). Furthermore, the thermal denaturation of *La*Fae revealed small changes up to a temperature of 45 °C and the melting temperature was found to be 56 °C (Supplementary Figure S2F).

### 2.3. Enzymatic Properties of LaFae

**Figure 2:**
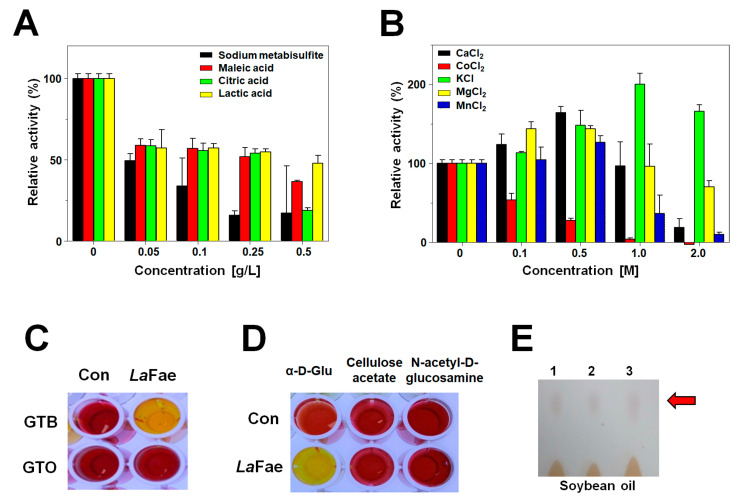
Enzymatic properties of *La*Fae. Effects of (**A**) organic acids, sodium metabisulfite and (**B**) inorganic salts on *La*Fae activity. The *La*Fae activity in the buffer alone was defined as 100%. All data are presented as mean ± standard deviation (s.d.) (*n* = 3). The hydrolysis of (**C**) lipids and (**D**) carbohydrates was investigated by pH-dependent assay using phenol red. (**E**) Synthesis of oleic acid esters from oil was investigated by thin-layer chromatography (Soybean oil with 1: methanol, 2: ethanol, 3: butanol). *La*Fae, *L. acidophilus* feruloyl esterase; GTB, glyceryl tributyrate; GTO, glyceryl trioleate; α-D-Gluc, α-D-glucose pentaacetate.

To evaluate the potential utility of *La*Fae as a fermentation agent, its activity in the presence of the organic acids, maleic acid, citric acid and lactic acid, as well as sodium metabisulfite was examined (Figure 2A). At 0.05 g/L sodium metabisulfite, *La*Fae retained approximately 50% of its initial activity, whereas at a 0.25 g/L concentration of the three organic acids, the enzyme showed approximately 60% of its original activity. These observations indicate that *La*Fae could be effectively used in the fermentation process [22]. Further analysis of *La*Fae activity in the presence of inorganic salts was performed by exposing the enzyme to different concentrations of CaCl_2_, CoCl_2_, KCl, MgCl_2_ and MnCl_2_ (Figure 2B). Results revealed that *La*Fae was highly activated in the presence of KCl, with approximately 180% of the initial activity being observed in response to treatment with 1.0 M. Moreover, when exposed to 2.0 M KCl, the activity of *La*Fae remained at 160% of its initial activity. Salt-induced activation was also detected with 0.5 M KCl, MgCl_2_, MnCl_2_ and CaCl_2_, respectively, with the enzyme exhibiting >150% of its initial enzyme activity when exposed to 0.5 M CaCl_2_. However, activity was almost completely lost in the presence of ≥1.0 M CoCl_2_. Thus, it seems that that a low ionic strength of cations enhances the activity of *La*Fae, while high-ionic strength decreases the activity, with the exception of CoCl_2_.

FAEs have a relatively broad substrate specificity in addition to the hydrolysis of HCA esters [14,23,24]. In the present study, we used a colorimetric assay to examine the hydrolytic properties of *La*Fae with respect to lipids and carbohydrates using a previously described method [25,26]. As shown in Figure 2C, of the two different types of lipids, glyceryl tributyrate GTB) and glyceryl trioleate (GTO), evaluated, substantial *La*Fae hydrolytic activity was detected only against GTB, as exhibited by the yellow color of the reaction mixture. *La*Fae activity on carbohydrate esters were examined using three substrates and displayed high activity only toward α-D-glucose pentaacetate (Figure 2D). In addition, *La*Fae can synthesize fatty acid esters using soybean oils and alcohols (methanol, ethanol and butanol) as substrates (Figure 2E). Biosynthesis of methyl, ethyl and butyl-oleic esters was detected using thin-layer chromatography, indicating that *La*Fae could be utilized to prepare biodiesels [24,27,28]. Collectively, our biochemical studies indicate that *La*Fae has promising hydrolytic/synthetic properties that could be harnessed for diverse commercial applications. 

Furthermore, we demonstrated that *La*Fae exhibits a hyperbolic kinetic behavior in the presence of different synthetic substrates. When *p*NB was used as a substrate, *V*_max_ and *K*_M_ values of 0.1 μM s^−1^ and 1.16 mM were obtained, respectively. Moreover, the catalytic efficiency for *p*NB (0.55 s^−1^ mM^−1^) was similar to that obtained for *p*NA (0.52 s^−1^ mM^−1^), indicating that *La*Fae essentially shows an equal affinity toward these two substrates (Supplementary Figure S3). These values are also comparable to those reported for other FAEs, such as Lj0536 [29].

### 2.4. Structural Analysis of LaFae

**Figure 3:**
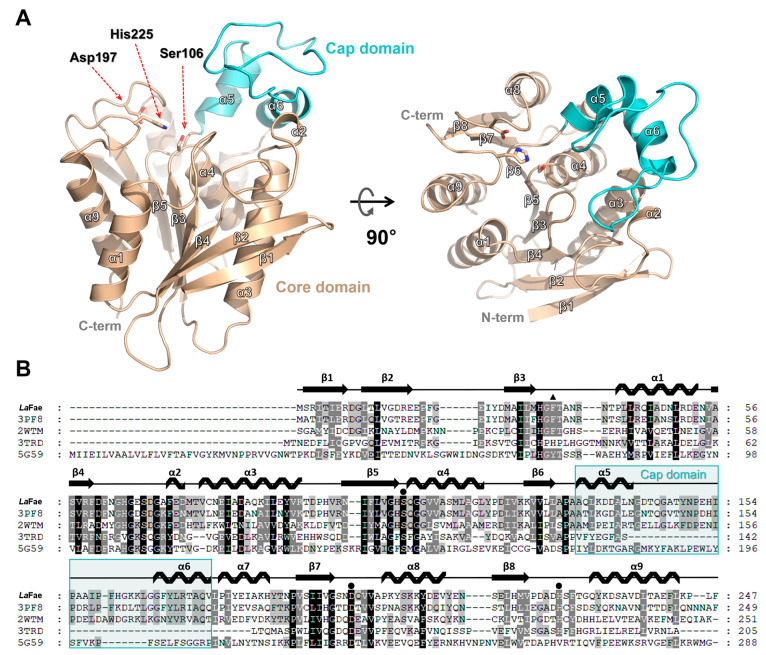
Overall structure and multiple sequence alignment of *La*Fae. (**A**) Cartoon representation of *La*Fae structure. The core domain and cap domain regions are colored wheat and aquamarine, respectively. The conserved catalytic triad of Ser106, Asp197 and His225 are shown using a stick model. (**B**) A structure-based multiple sequence alignment of *La*Fae with cinnamoyl esterase LJ0536 from *Lactobacillus johnsonii* (UniProtKB code D3YEX6; PDB code 3PF8), Est1E from *Butyrivibrio proteoclasticus* (UniProtKB code D2YW37; PDB code 2WTM), an α-β serine hydrolase homologue from *Coxiella burnetii* (UniProtKB code Q83AV9; PDB code 3TRD) and Esterase Pf2001 from *Pyrococcus furiosus* (UniProtKB code Q8TZJ1; PDB code 5G59). The conservation scores of amino acids are represented using a color spectrum ranging from black (indicating high conservation) to white (indicating low conservation). The catalytic triad residues (Ser106, Asp197 and His225) are indicated with solid black circles and the residue (Phe34) that affects activity is indicated with a black triangle. The cap domain region of *La*Fae is indicated by a cyan box. *La*Fae, *L. acidophilus* feruloyl esterase.

The unliganded crystal structure of *La*Fae from *L. acidophilus* was determined at 2.3 Å resolution (Figure 3A and Table 1 using the molecular replacement method, which used the crystal structure of cinnamoyl esterase from *L. johnsonii* (PDB code 3PF8; 70% sequence identity) as a template model. The crystal of unliganded *La*Fae belongs to the space group of *P*2_1_2_1_2_1_ with two molecules in the asymmetric unit. The crystal structure of *La*Fae contains nine α helices and eight β strands. The β1 and β2 strands form an antiparallel β-sheet while the β3–β8 strands form parallel β-sheets. Seven α-helices surround the β-sheets and form a core domain that contains an active site with conserved catalytic triad residues (Ser106, Asp197 and His225). The α5- and α6-helices form a cap domain (from Ala133 to Val175) (Figure 3B). These structural features showed a canonical α/β fold of esterases. Structural homolog search using the DALI server [30] showed that cinnamoyl esterase from *L. johnsonii* (PDB code 3PF8) had the highest structural similarity with a Z-core of 42.1. In addition, Est1E from *Butyrivibrio proteoclasticus* (PDB code 2WTM) [31], monoglyceride lipase from *Mycobacterium tuberculosis* (PDB code 6EIC) [32] and putative hydrolase from *Agrobacterium vitis* (PDB code 3LLC) showed considerable structural similarities with the *La*Fae structure (Supplementary Table S1).

**Figure 4:**
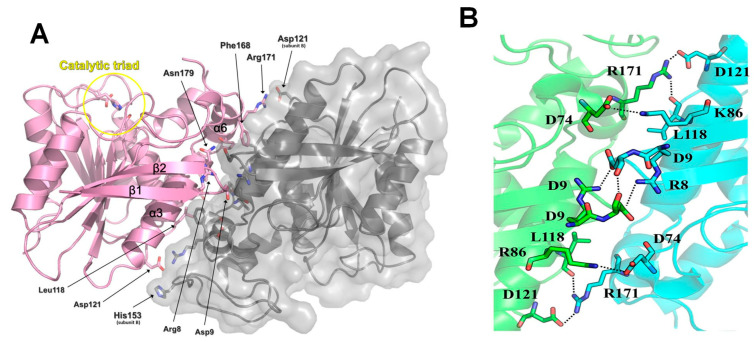
Dimerization of *La*Fae. (**A**) The α3, α6 and β1-β2 hairpin regions are involved in the dimerization interface. Although several salt bridges and hydrogen bonds participate in dimerization interaction, hydrophobic interactions are predominant. Subunit A is represented in the illustration with a pink color. Subunit B is presented in the surface representation model with gray color. The residues involved in dimerization interactions are shown using a stick model. The catalytic-triad-containing active site is shown within the yellow circle. (**B**) Close-up view of residue interaction at dimerization interface of *La*Fae.

Two protomers of *La*Fae have been identified in the symmetric unit (Figure 4A) during the crystal structure determination. In the *La*Fae crystal structure, the α3, α4, α6 and α7 helices, as well as the β1-β2 hairpin, are involved in the dimerization interface. Several hydrogen bonds (Asp9-Leu11, Asp94-Lys86 and Leu118-Arg171) and salt bridges (Arg8-Asp9, Asp74-Lys86, Asp121-Arg171 and Asp121-His153) were observed at the dimerization interface; however, hydrophobic interactions (Ile81, Ala82, Leu118, Phe168, Val175, Leu176, Pro177 and Ile181) were predominant during the dimerization of *La*Fae (Figure 4B). Similar composition of reisues and the dimerization was also found in the cinnamoyl esterase from *L*. *johnsonii* (PDB code 3PF8) and Est1E from *B*. *proteoclasticus* (Supplementary Table S1).

**Figure 5:**
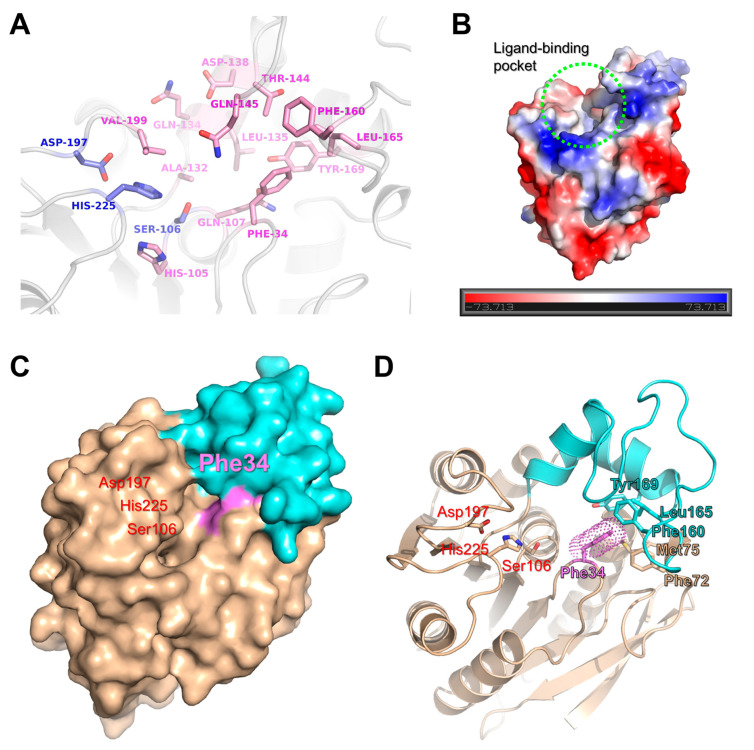
Active site of *La*Fae. (**A**) Close-up view of the active site and ligand-binding pocket of *La*Fae. The catalytic-triad residues and ligand-binding, pocket-forming residues are shown using a stick model colored slate and pink, respectively. (**B**) Electrostatic surface potential map of *La*Fae shows a positively charged environment of the active site. The ligand-binding pocket is indicated by the dotted light-green circle. (**C**) Surface view of *La*Fae. The core domain and cap domain are colored in wheat and aquamarine, respectively. The residue Phe34 is colored violet. (**D**) The hydrophobic interactions between the core domain and cap domain. The residue Phe34 occupies space within the ligand-binding pocket. Residues involved in hydrophobic interaction between two domains are shown using a stick representation. *La*Fae, *L. acidophilus* feruloyl esterase.

The active site of *La*Fae is located within the core domain. The substrate binding pocket is comprised of the β3-α1 loop, β7-α8 loop and β8-α9 loop regions (Figure 5A). In detail, several polar residues (His105, Gln107, Gln134, Asp138, Thr144, Gln145 and Tyr169) and hydrophobic residues (Phe34, Ala132, Leu135, Phe160, Leu165 and Val199) have been shown to form the ligand-binding pocket. Furthermore, the conserved catalytic triad of Ser106, Asp197 and His225 was also observed to be located in this pocket. The residue Ser106 is located at the end of the β5 strand and the residues Asp167 and His225 are located on the β7-α8 loop and the β8-α9 loop, respectively. The surface surrounding the ligand-binding pocket is positively charged, presumably to attract negatively charged substrates, such as acyl group-containing substrates (Figure 5B). In addition, the cap domain was observed to be located above the ligand-binding site and this domain was hypothesized to have a gatekeeper role for substrate binding and release of the reaction product.

**Figure 6:**
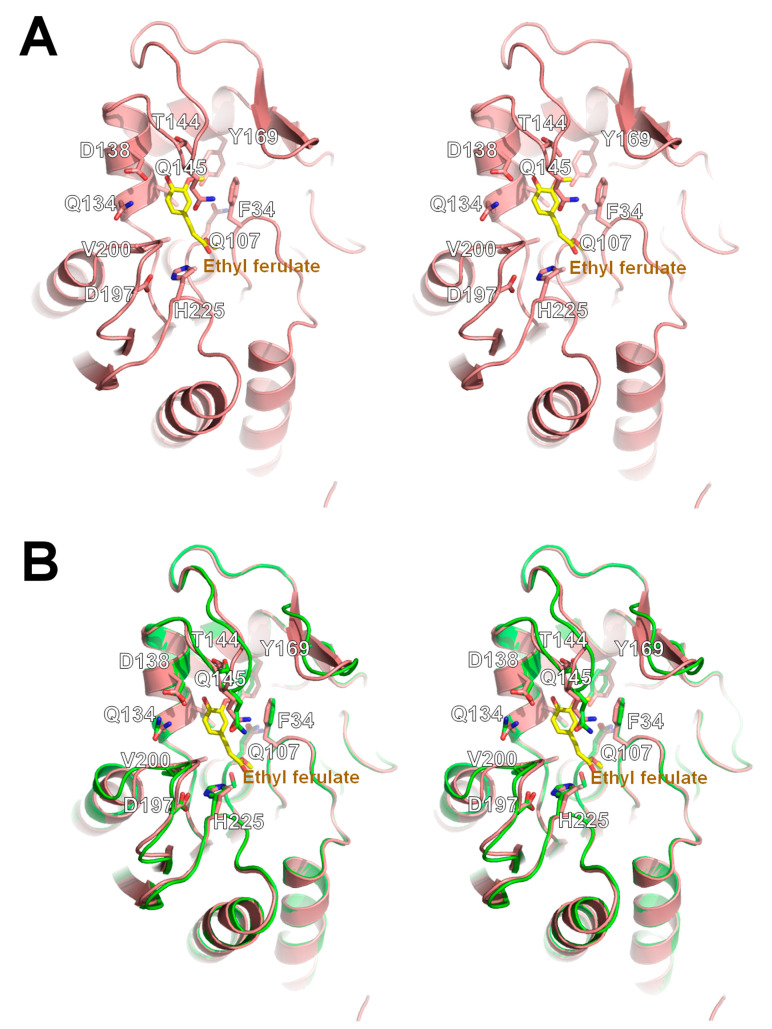
Analysis of the molecular structure of ethyl ferulate bound *La*Fae. (**A**) Stereo view of the overall structure of ethyl ferulate bound *La*Fae. (**B**) Stereo view of superimposed structures of unliganded (green) and ethyl ferulate bound (salmon) *La*Fae. The interaction residues with ethyl ferulate are shown using a stick model.

**Figure 7:**
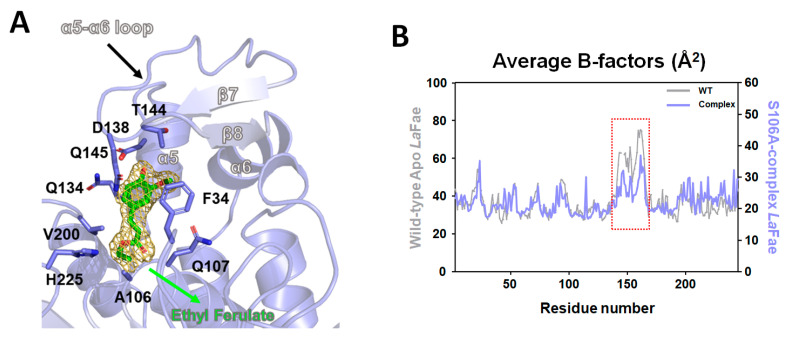
Substrate binding mode of *La*Fae and b-factor analysis. (**A**) The electron-density map (contoured at 1.0 σ) for ethyl ferulate is shown in mesh (blue). Interaction residues are shown in a stick model. (**B**) Comparative b-factor analysis toward unliganded (gray) and liganded (slate) *La*Fae structure. The red dashed square represents the average B-factors of α5- α6 loop region.

We also resolved the crystal structure of ethyl ferulate-bound *La*Fae mutant (S106A) at 2.19 Å resolution and analyzed its substrate binding mode. Ethyl ferulate complexed *La*Fae crystallized in the *C*121 space group with four molecules in an asymmetric unit. The substrate-binding pocket of *La*Fae comprised of hydrophobic residues F34 and V200 and polar residues Q107, Q134, D138, T144, Q145, Y169, D197 and H225 (Figure 6A). A structural superposition of the ligand-free form and ethyl ferulate-bound *La*Fae yielded a root-mean-square deviation value of 0.261 Å over 209 residues, indicating a highly similar overall structure (Figure 6B). In a close-up view of the substrate binding site of the complex structure, the electron density map (contoured at 1.0 σ) of ethyl ferulate was observed near the S106A mutation position (Figure 7A). Notably, the carbonyl group of ethyl ferulate was discovered to interact directly with the NE2 atom of His225 at a 2.5 Å distance and the 4′-hydroxyl group of ethyl ferulate also formed hydrogen bonds with Asp138 at a 2.4 Å distance (Figure 7A). In the substrate-bound conformation, two anti-parallel β strands formed a sheet within the cap domain (α5–α6 loop region of the apo structure). Additionally, the comparative b-factor analysis showed that the binding of ethyl ferulate caused a reduced structural flexibility on the α5–α6 loop region (Figure 7B). It is thought that this loop region plays a role in the open/close movement of *La*Fae when the substrate is bound.

### 2.5. Mutational Analyses of LaFae

**Figure 8:**
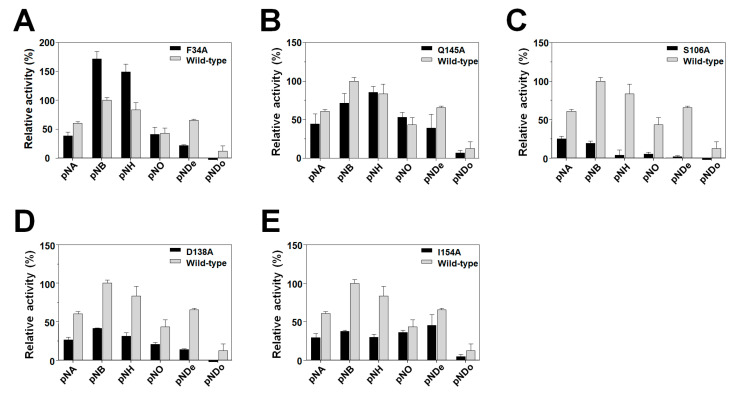
Substrate specificity of *La*Fae mutants. Enzymatic assay results for (**A**) F34A, (**B**) Q145A, (**C**) S106A, (**D**) D138A and (**E**) I154A are shown. Relative activity of wild-type *La*Fae against *p*NB were defined as 100%. All the results from mutants (black) were compared to the wild-type *La*Fae (gray). All data are presented as mean ± standard deviation (s.d.) (*n* = 3). *La*Fae, *L. acidophilus* feruloyl esterase; *p*NB, *p*-nitrophenyl butyrate.

To investigate the roles of the aforementioned amino acids, site-directed mutagenesis was performed to generate six mutants (F34A, S106A, D138A, Q145A and I154A). Following expression and purification, the enzymatic properties of these mutants were examined using *p*-nitrophenyl esters of different lengths from C2 to C16. In line with our expectations, we observed that the activity of the S106A mutant was almost lost, whereas there were notable reductions in the activities of D138A and I154A mutants (Figure 8C–E). In contrast, compared to wild-type *La*Fae, F34A exhibited noticeably enhanced activities (Figure 8A) of an approximate 1.6-fold increase for *p*NB, whereas three other mutants (D138A and I154A) retained only about 30% of the wild-type activity for the same substrate. Furthermore, Q145A retained approximately 100% of the activity exhibited by wild-type *La*Fae for *p*-nitrophenyl hexanoate as a substrate (Figure 8B). With respect to the activity of the F34A mutant, we hypothesize that the bulky nature of the Phe side chain enhances binding to short-chain substrates. Within the *La*Fae structure, Phe34 forms hydrophobic interactions with the Phe72, Met75, Phe160, Leu165 and Tyr169 residues and conceivably stabilizes and controls the movement of the cap domain via hydrophobic interactions. Thus, the F34A mutation may confer a greater cap domain flexibility, as well as a broader substrate binding space. In summary, these mutants could regulate the ferulic acid activity of *La*Fae, thereby highlighting the important functional roles of these amino acids in catalysis.

### 2.6. Ferulic Acid Production Using LaFae

**Figure 9:**
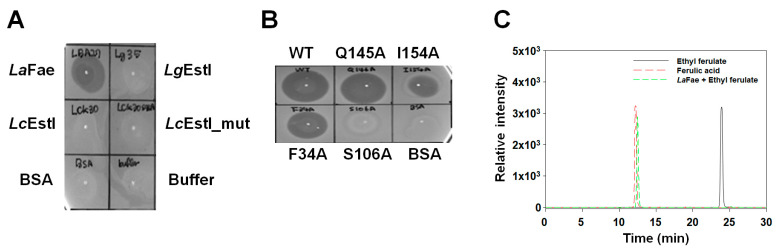
Feruloyl esterase activity of *La*Fae. Ferulic acid production of (**A**) *La*Fae and other enzymes derived from LAB strain (*Lg*EstI, esterase from *Lactococcus garvieae*; *Lc*EstI, esterase from *Leuconostoc citreum*). Note that only *La*Fae led to the production of ferulic acid in the plate assay. (**B**) Effects of *La*Fae mutants on ferulic acid production. (**C**) Analysis of ferulic acid production from ethyl ferulate using HPLC. *La*Fae, *L. acidophilus* feruloyl esterase; HPLC, high-performance liquid chromatography.

The ability of *La*Fae to release ferulic acids from plant biomass was investigated using de-starched wheat bran (DSWB), de-starched rice bran (DSRB) and corn stalks (CSs). Initially, we conducted a plate assay in which *La*Fae was used to hydrolyze ethyl ferulate on an agar plate. The appearance of halo rings indicated the presence of FAE activity in the different enzymes screened. Although we screened several enzymes derived from LAB for FAE activity, only *La*Fae was observed to form a large clear zone of hydrolysis on the opaque plates containing ethyl ferulate in medium (Figure 9A). Similar hydrolysis was obtained for the F34A and Q145A mutants, whereas the I154A mutant produced a notably smaller hydrolysis ring zone (Figure 9B). Furthermore, HPLC analysis showed that the retention time of *La*Fae reaction mixture with ethyl ferulate corresponds with ferulic acid standard (Figure 9C).

To evaluate the amounts of ferulic acid from agricultural waste, we initially determined the contents of ferulic acid in DSWB, DSRB and CSs following alkaline hydrolysis with sodium hydroxide, which yielded 3.5, 2.9 and 2.7 mg g^−1^, respectively (Supplementary Figure S4). Following incubation with *La*Fae alone, only relatively small amounts of ferulic acid were released from DSWB (6.3% of that released via chemical extraction), DSRB (3.1%) and CSs (2.9%). In previous studies, it was shown that a combination of xylanase and FAE can enhance the release of ferulic acid [10,33,34]. Therefore, we examined the efficacy of the combination of *La*Fae and xylanase, derived from the fungus *Aspergillus niger*. We found that the amount of ferulic acid produced in response to the combined treatment with *La*Fae and xylanase was approximately 6–10 times higher than that of *La*Fae alone. Specifically, 37.7%, 34.8% and 42.2% higher amounts of ferulic acid were produced from wheat bran, rice bran and CSs, respectively.

## 3. Materials and Methods

### 3.1. Chemicals and Reagents

All DNA modification enzymes were purchased from Takara Korea Biomedicals (Seoul, Republic of Korea). Nucleic acid preparation kits were obtained from GE Healthcare (Seoul, Republic of Korea) and Qiagen (Seoul, Republic of Korea). Other chemicals were obtained from Sigma-Aldrich (Seoul, Republic of Korea).

### 3.2. Preparation of Wild-Type and Mutants of LaFae

*L. acidophilus* NCFM (KCTC 3145) was cultured in deMan Rogosa Sharpe medium. The *La*Fae gene was obtained using polymerase chain reaction (PCR) from *L. acidophilus* chromosomal DNA. The final product was inserted into a pET-21a vector using BamHI and XhoI restriction enzymes and subsequently introduced into *Escherichia coli* BL21 (DE3) for heterologous expression of His_6_-tagged *La*Fae. Site-directed mutagenesis was performed and all mutants obtained (F34A, S106A, D138A, Q145A and I154A) were confirmed through DNA sequencing. Primers used to generate the clone and mutants are listed in Supplementary Table S2.

Recombinant *E. coli* BL21 (DE3) were cultured in LB medium containing ampicillin (100 μg/mL) to an OD_600nm_ of 0.6 at 37 °C. Subsequently, expression of the recombinant *La*Fae was induced by addition of 1 mM of isopropyl-β-D-1-thiogalactosideand incubation for 4 h, after which cells were harvested through centrifugation at 2000 rpm for 15 min. Cell disruption was carried out by sonication in the lysis buffer (50 mM NaH_2_PO_4_, 300 mM NaCl, 30 mM Imidazole). The supernatant was separated by centrifugation at 16,000 rpm for 40 min and loaded onto a HisTrap column (GE Healthcare, Amersham, UK). The recombinant *La*Fae protein was subsequently eluted with the elution buffer (50 mM NaH_2_PO_4_, 300 mM NaCl, 300 mM Imidazole). Subsequent purification and buffer exchange were conducted through size-exclusion chromatography using the protein storage buffer (20 mM Tris-HCl, pH 8.5) and the purity of the protein was assessed using SDS-PAGE.

### 3.3. Oligomerization of LaFae

Oligomerization of *La*Fae in solution was investigated by gel-filtration chromatography and analytical ultracentrifugation (AUC). For gel-filtration chromatography, the protein standard mix, including cytochrome C (12.4 kDa), carbonic anhydrase (29 kDa), albumin (66 kDa), alcohol dehydrogenase (150 kDa) and β-amylase (200 kDa) was eluted in 20 mM Tris-HCl (pH 8.0) using HiPrep S-200R column and the standard curve was generated. The *La*Fae was eluted using the same method described above and the molecular weight in the solution was calculated. The AUC was performed in 20 mM Tris-HCl (pH 8.0) buffer using a ProteomeLab XL-A (Beckman Coulter, Carlsbad, CA, USA). The *La*Fae was centrifuged at 14,225× *g* for 10 min. The sedimentation profile monitored at 280 nm was analyzed using the SEDFIT program. Coomassie Brilliant Blue R-250 and 4-methylumbelliferone acetate were used for activity staining during the protein electrophoresis analysis [35]. Briefly, the overlay activity assay was conducted using native-PAGE analysis. The resulting gel was then washed three times using a storage buffer. After the washing steps, the gel was incubated with 4-methylumbelliferone acetate, allowing the detection of a fluorescent signal under UV illumination.

### 3.4. Measurement of Esterase Activity

The substrate specificity of wild-type *La*Fae and generated mutants was investigated in 96-well microplate (Corning, Costar, NY, USA) using Epoch2 microplate spectrophotometer (BioTek, Winooski, VT, USA). The different chain lengths of *p*-nitrophenyl (*p*NP) esters, including *p*NP acetate (C2, *p*NA), *p*NP butyrate (C4, *p*NB), *p*NP hexanoate (C6, *p*NH), *p*NP octanoate (C8, *p*NO), *p*NP decanoate (C10, *p*NDe), *p*NP dodecanoate (C12, *p*NDo) and *p*NP palmitate (C16, *p*NPp) were used as substrates. The released *p*-nitrophenol was measured at 405 nm in triplicate. The *La*Fae activity against *p*-nitrophenyl butyrate was defined as 100%.

### 3.5. Chemical Stability Assay

The organic solvents, ethanol, isopropyl alcohol, sodium dodecyl sulfate (SDS), Triton-X100 and Tween-20 were used to examine the chemical stability of *La*Fae. Additionally, the enzymatic activities of *La*Fae in 0–2 M of NaCl, 5–40% of glycerol and 0–3 M of urea solution were investigated to assess its industrial application potential. The *La*Fae activity was estimated after incubation for 1 h in the respective solutions and the activity of *La*Fae in the storage buffer alone was considered as 100%. Additionally, the effects of various types of acids and salts on *La*Fae was estimated after 1 h incubation in 0, 0.05, 0.1, 0.25, 0.5 g/L of three types of acid (maleic acid, citric acid, lactic acid) and 0, 0.1, 0.5, 1.0, 2.0 M of five types of salt (CaCl_2_, CoCl_2_, KCl, MgCl_2_, MnCl_2_) using an Epoch 2 Microplate Spectrophotometer (BioTek, Winooski, VT, USA). The activity of *La*Fae using *p*-nitrophenyl butyrate as substrate in buffer alone was set as 100% of relative activity and all experiments were performed in triplicate. The mean ± standard deviation (s.d.) (*n* = 3) were calculated and presented in figures.

### 3.6. Thermal Stability and Circular Dichroism Analysis

The effect of temperature on the stability of *La*Fae was examined by incubating the enzyme at temperatures between 25 and 60 °C and measuring the residual activity for 1 h, at 15 min intervals. While measurements were performed in triplicate, the *p*-nitrophenyl butyrate was used as substrate and the activity at 25 °C was set as 100%. Far-ultraviolet (UV) circular dichroism (CD) analysis was performed to monitor the structural denaturation profile with increasing temperature. Data collection was carried out in 1 mm pathlength cell with a 0.5 nm bandwidth and 1 s response time. An average of three accumulations was used to generate the final spectra. The spectra were obtained at 190–240 nm (Jasco, Tokyo, Japan) to evaluate the secondary structure of *La*Fae, and the thermal unfolding profile was monitored from 25 to 80 °C at 222 nm in a thermostatic cell holder.

### 3.7. Hydrolytic Activity Assay

To examine the hydrolytic activity of *La*Fae on lipids and carbohydrate substrates, a pH- indicator-based colorimetric assay was performed on a microplate [25,27]. Glyceryl tributyrate and trioleate were used as lipid-type substrates and α-D-glucose pentaacetate, cellulose acetate and *N*-acetyl-glucosamine were used as acetylated carbohydrate substrates. In the phenol-red containing solution of each substrate, 100 μg *La*Fae enzyme was added to start a hydrolytic reaction. As a result of the pH shift assay of *La*Fae, the hydrolysis reaction changed the color of the phenol-red containing mixture to yellow. For the synthesis of oleyl esters, three types of alcohols (methanol, ethanol and 1-butanol) were incubated with soybean oil and 100 μg *La*Fae by continuous shaking at 37 °C, and reaction products were analyzed by thin-layer chromatography.

### 3.8. Crystallization and Data Collection

The wild-type apo and S106A-ethyl ferulate complex of *La*Fae were crystallized by the sitting-drop vapor-diffusion method at 296 K using a mosquito crystallization robot (TTP LabTech, Melbourn, UK). Commercially available crystallization screening solution kits were used, including MCSG I-IV, MIDAS, Morpheus (Molecular Dimensions, Rotherham, UK), Index and SaltRx (Hampton Research, Aliso Viejo, CA, USA). Each crystallization drop was set with 300 nL protein solution and 300 nL reservoir solution and equilibrated against an 80 μL reservoir solution at 296 K. The optimized crystals of wild-type apo *La*Fae were observed using 0.1 M Bis-Tris:HCl at pH 6.5 and 20% (*w*/*v*) PEG MME 5000 (MCSG Ⅰ #F1) after 1–2 d. For co-crystallization of the S106A mutant with substrate, ethyl ferulate was dissolved in ethanol. The molar ratio of protein to ligand was 1:10 at a final ligand concentration of 3.5 mM. The complex crystals of S106A-ethyl ferulate were observed after 4 d in optimized conditions of 0.02 M magnesium chloride, 0.1 M HEPES:NaOH, pH 7.5 and 22% (*w*/*v*) polyacrylic acid 5100 (MCSG ⅠI #F5), using hanging-drop vapor-diffusion method at 296 K. The single crystal of wild-type *La*Fae was mounted without cryoprotectant and the S106A-ethyl ferulate complex crystal was mounted after being gently soaked in 20% glycerol mixed in a crystallization reservoir solution as cryoprotectant. X-ray diffraction data were collected from crystals flash-frozen at 193 K under liquid nitrogen at BL-5C beam line of the Pohang Accelerator Laboratory (PAL, Pohang, Republic of Korea) with an oscillation of 1° per image. A total of 200 and 360 diffraction images of the wild-type and the S106A-ethyl ferulate complex crystals were collected, respectively. Each data set was indexed, integrated and scaled using the HKL-2000 software [36]. The detailed X-ray diffraction data collection statistics are shown in Table 1.

### 3.9. Structure Determination and Refinement

The crystal structures of wild-type apo and S106A-ethyl ferulate complex of *La*Fae were determined at 2.3 Å and 2.19 Å resolution, respectively. The phase problem was solved through the molecular replacement method using the MOLREP program from the CCP4i suite [37]. The crystal structure of cinnamoyl esterase from *Lactobacillus johnsonii* (PDB code 3PF8), which shares 70% sequence identity with *La*Fae, was used as a search model to define the apo structure of *La*Fae. The refined apo structure was used as a template for the structural analysis of S106A-ethyl ferulate complex. During the manual model building of the S106A-ethyl ferulate complex structure, all ligands shown on each chain were modeled with an occupancy of 1.0. The initial models of both apo and complex structures were iteratively rebuilt and refined using Coot [38], REFMAC [39] and phenix.refine programs [40]. The final structures exhibited *R*_cryst_ and *R*_free_ values of 0.227 and 0.280 in apo and 0.186 and 0.213 in complex structures, respectively. Validation of both final models was carried out using MolPorbity [41]. Structure refinement statistics are listed in Table 1. All structural figures were generated using the PyMOL molecular-graphics system [42]. The atomic coordinate and structure factor of wild-type apo and S106A-ethyl ferulate complex of *La*Fae have been deposited in the Protein Data Bank under accession codes 7XRH and 7XRI, respectively.

### 3.10. Determination of LaFae Feruloyl Esterase Activity

To verify FAE activity, the plate assay was performed at 37 °C, as previously described [43]. The circular zones were generated as a result of ethyl ferulate hydrolysis. Previously characterized enzymes were used to examine feruloyl esterase activity [44]. *La*Fae activity was estimated using ethyl ferulate (1 mM) as a substrate; 10 µL of 1 mg/mL *La*Fae was added to 300 µL of reaction mixture. After incubation, the mixture was analyzed using an Eclipse Plus RR-C18 column with a 1220 Infinity II LC system (Agilent, Santa Clara, CA, USA). The samples were separated in a mobile phase of acetonitrile: water (80:20) at 30 °C [45].

To produce ferulic acid from agricultural biomass by *La*Fae, wheat bran, rice bran and corn stalks were firstly de-starched by incubation with 1 M NaOH at 70 °C. The extraction of ethyl ferulate was performed in an 800 µL reaction mixture containing 20 mg of de-starched wheat bran, rice bran and corn stalks and 5 mg *La*Fae in 20 mM Tris-HCl (pH 7.5) and 150 mM NaCl [46,47]. To investigate the synergism between *La*Fae and xylanase, we analyzed the amounts of ferulic acid produced after incubation of substrates with *La*Fae and *A. niger* xylanase (Megazyme, Bray, Ireland). The amounts of released ferulic acid were measured using HPLC, as described above. All experiments were performed in triplicate and each error rate was calculated and presented in figures.

## 4. Conclusions

Herein, we provide a comprehensive characterization of a universal feruloyl acid esterase (*La*Fae) from LAB, and its biotechnological applications in ferulic acid production from plant biomass. Several residues surrounding the active site were mutated to identify the catalytically important residues for substrate binding and specificity of *La*Fae and their activities relative to the wild-type were measured. Interestingly, the F34A mutant exhibited relatively increased catalytic activities against *p*NB and *p*NH. Since it is thought that the Phe34 plays a role of physically supporting and connecting the cap domain through hydrophobic interactions, the F34A mutation may provide a more flexible cap domain movement, as well as a broader substrate binding space. Furthermore, the conformational change of the cap domain region in substrate-free, as well as the ethyl ferulate bound structure, presented open and closed movements of *La*Fae when harboring ethyl ferulate as a substrate. Additionally, we showed that *La*Fae from the probiotic bacterium *L. acidophilus* can be directly used to enhance the yields of high-value hydroxycinnamates from natural resources. Thus, we believe that *La*Fae will have potential applications in foods, cosmetics, animal feed and biodiesel industries.

## Figures and Tables

**Table 1:** X-ray diffraction data collection and refinement statistics.

Data Collection	*La*Fae	S106A Complex with Ethyl Ferulate
X-ray source	BL-5C beam line	BL-5C beam line
Space group	*P*2_1_2_1_2_1_	*C*121
Unit-cell parameters (Å, °)	a = 49.2, b = 74.6, c = 123.4, α = β = γ = 90	a = 130.4, b = 153.4, c = 91.3, α = 90, β = 127.4, γ = 90
Wavelength (Å)	0.9794	0.9794
Resolution (Å)	50.00–2.30 (2.34–2.30)	50.00–2.19 (2.23–2.19)
Total reflections	137,499	515,156
Unique reflections	20,175 (1031)	71,881 (3569)
Average I/σ (I)	25.06 (4.62)	29.54 (6.93)
*R*_merge_ ^a^	0.198 (0.506)	0.106 (0.388)
Redundancy	6.8 (7.2)	7.2 (6.6)
Completeness (%)	97.2 (100.0)	98.9 (98.2)
Refinement		
Resolution range (Å)	38.96–2.30 (2.38–2.30)	44.53–2.19 (2.27–2.19)
No. of reflections of working set	20,108 (1845)	71,872 (6767)
No. of reflections of test set	2000 (183)	3603 (319)
No. atoms		
Protein	3624	7868
Ligands	N/A	64
Solvent	66	440
*R*_cryst_ ^b^	0.227 (0.251)	0.186 (0.192)
*R*_free_ ^c^	0.280 (0.314)	0.213 (0.224)
R.m.s. bond length (Å)	0.012	0.019
R.m.s. bond angle (°)	1.324	1.93
Average B value (Å^2^)		
Protein	40.04	23.7
Ligand	N/A	27.84
Solvent	39.06	28.61
Ramachandran plot		
Favored (%)	95.9	95.82
Allowed (%)	4.2	4.08
Outliers (%)	0.0	0.1

^a^*R*_merge_ = ∑|< I > − I|/∑ < I >. ^b^ *R*_cryst_ = ∑||Fo| − |Fc||/∑|Fo|. ^c^ *R*_free_ calculated with 5% of all reflections excluded from refinement stages using high-resolution data. Values in parentheses refer to the highest resolution shells.

## Data Availability

Wild-type apo and S106A-ethyl ferulate complex of *La*Fae have been deposited in the Protein Data Bank under accession codes 7XRH and 7XRI, respectively.

## Supplementary Materials

The supporting information can be downloaded at: https://www.mdpi.com/article/10.3390/ijms241311170/s1. References [29,31,32,48,49] are cited in the supplementary materials.
