# Supplementary material for: Feruloyl Esterase (LaFae) from Lactobacillus acidophilus: Structural Insights and Functional Characterization for Application in Ferulic Acid Production
Source: Int J Mol Sci. 2023 Jul 6;24(13):11170. doi: 10.3390/ijms241311170 (PMC10342849; doi:10.3390/ijms241311170)
Supplement: Supplementary file 1 [file ijms-24-11170-s001.zip › ijms-2410694-supplementary.pdf]

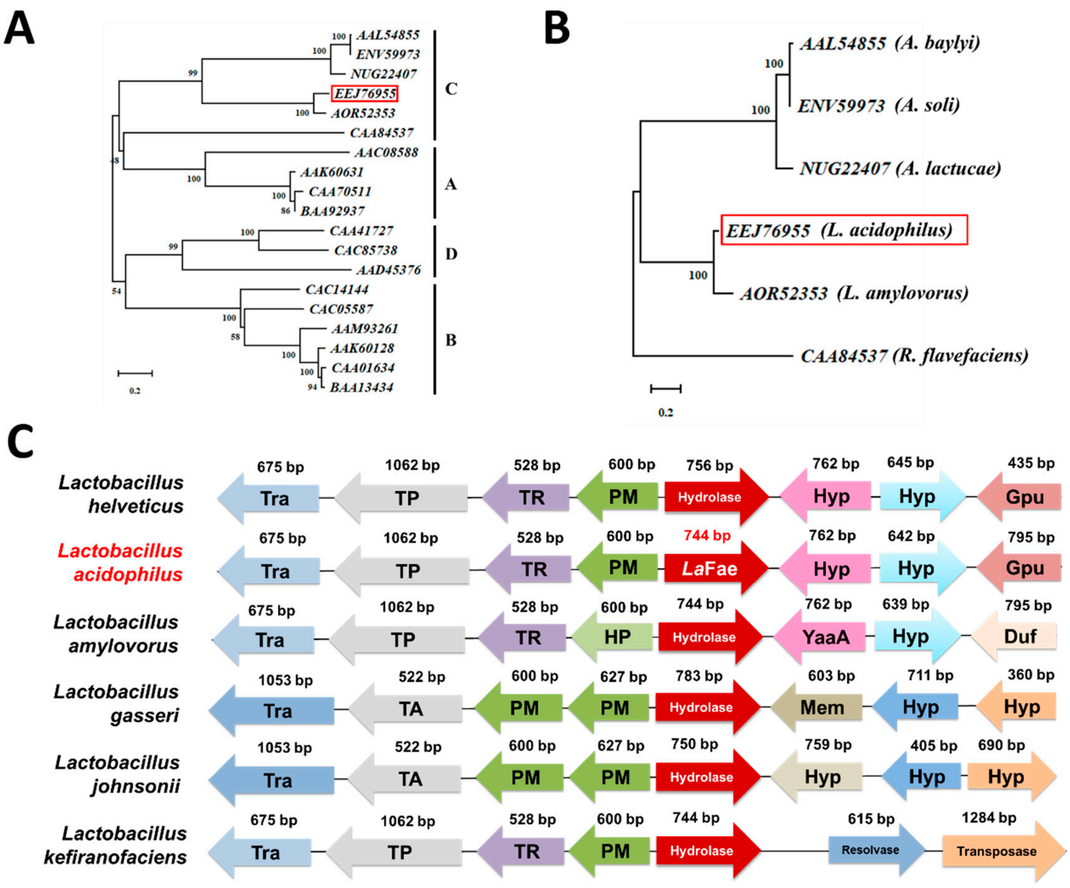

Supplementary Figure S1. The phylogenetic and gene cluster analysis of *La*Fae. (A) The phylogenetic tree of *La*Fae (NCBI accession code: EEJ76955.1) classifies it as type C feruloyl esterases. **(B)** The FaeLam from *Lactobacillus amylovorus* is presented as most similar enzyme to *La*Fae. **(C)** Gene cluster analysis between *Lactobacillus* species. Tra: ABC transporter ATP-binding protein, TP: ABC transporter permease protein, TR: Transcription regulator, PM: Phosphoglycerate mutase, HP: Histidine phosphatase family protein, Hyp: Hypothetical protein, GPU: Galactose-1-phosphate uridylyltransferase, YaaA: Peroxide stress resistance protein, and Duf: Domains of unknown function.

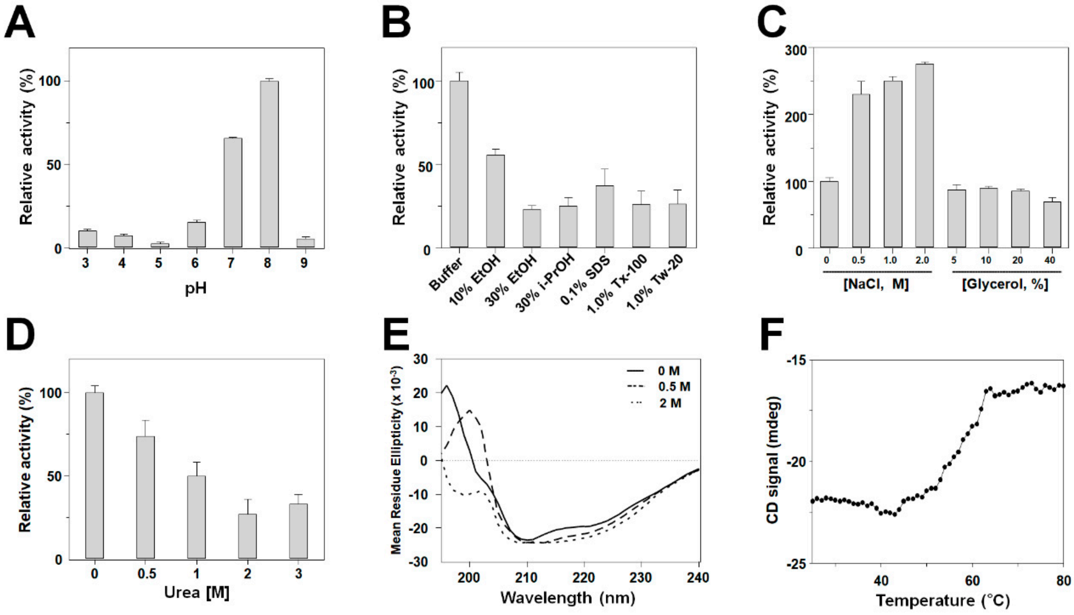

Supplementary Figure S2. Enzymatic activity of *La*Fae under various conditions and CD analysis. **(A)** Optimal pH for *La*Fae activity. **(B)** Effects of organic ethanol, 1-propanol, SDS, Triton X-100, and Tween-20 on *La*Fae activity. **(C)** Effects of increasing NaCl and glycerol concentration on *La*Fae activity. **(D)** Effects of increasing urea concentration on *La*Fae activity. **(E)** CD spectra of *La*Fae in 0, 0.5, and 2 M urea solution. **(F)** Thermal denaturation curve of *La*Fae. Melting temperature (Tm) was calculated to be 56 °C.

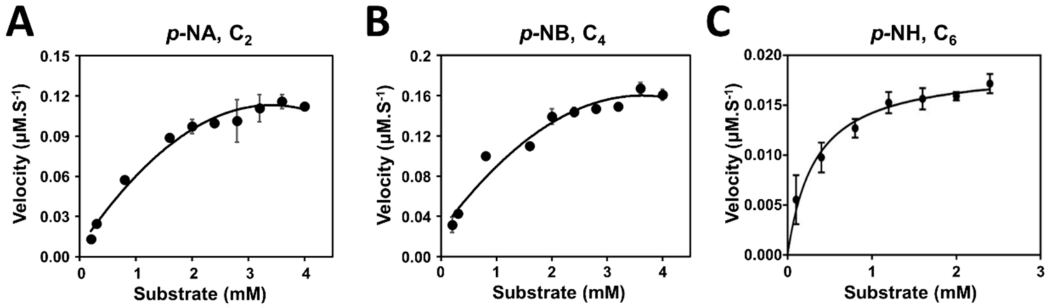

D

Substrates	V_{max} (μMs^{-1})	K_M (mM)	k_{cat} (s^{-1})	k_{cat}/K_M (s^{-1}mM^{-1})
p-NA, C_2	0.16 ± 0.07	1.50 ± 0.15	0.54 ± 0.04	0.36
p-NB, C_4	0.20 ± 0.02	0.97 ± 0.14	0.66 ± 0.02	0.67
p-NH, C_6	0.02 ± 0.00	0.38 ± 0.06	0.12 ± 0.01	0.32

Supplementary Figure S3. Kinetics assay of *La*Fae. Enzyme kinetics assay was performed using *p*-NA, C2 **(A),** *p*-NB, C4 **(B),** *p*-NH, C6 **(C).** **(D)** V_{max}, K_M, and K_{cat} values of *La*Fae against three different lengths substrates are calculated and summarized in the table**.**

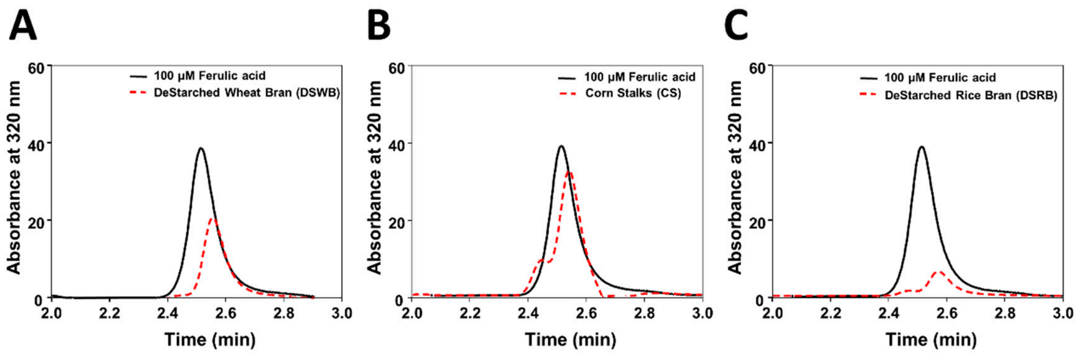

Supplementary Figure S4. Amount of ferulic acid from de-starched wheat bran (DSWB), corn stalks, and de-starched rice bran (DSRB). 100 µM solution of ferulic acid was used as standard (black). The amount of ferulic acid from DSWB **(A),** corn stalks **(B),** and DSRB **(C)** are presented as red dotted line.

Supplementary Table S1. Structural homologues of *La*Fae from DALI search.

**Protein**	**PDB Code**	**DALI Z-Score**	**Sequence % Identities**	**Oligomeric state in solution**	**Reference**
Cinnamoyl esterase LJ0536 from *Lactobacillus johnsonii*	3PF8	42.1	70	Dimer	[1]
Est1E from *Butyrivibrio proteoclasticus*	2WTM	37.6	30	Dimer	[2]
Monoglyceride lipase from *Mycobacterium tuberculosis*	6EIC	28.1	18	Monomer	[3]
Putative hydrolase from *Agrobacterium*	3LLC	27.6	21	N/A	Not yet published
Dienelactone hydrolase from *Anabaena variabilis* ATCC 29413	2O2G	27.1	21	N/A	Not yet published
YcjY from *E. coli*	5XB6	27.1	18	N/A	Not yet published
Monoglyceride lipase form human	3HJU	26.4	18	Dimer	[4]
Putative alpha/beta hydrolase from *Agrobacterium tumefaciens*	2I3D	26.1	20	N/A	Not yet published
alpha-beta serine hydrolase homologue from *Coxiella burnetii*	3TRD	25.9	22	N/A	[5]

Supplementary Table S2. Primer sequences used to generate a clone and *La*Fae mutants.

Primers	Sequences (5` to 3`)
Cloning forward	GATTTGGATCCATGTCTCGCATTACAA
Cloning reverse	GCATTCTCGAGAATTTTATGGGGCTTC
F34A forward	CTTATGCATGGCGCGACTGCAAACAGA
F34A reverse	TCTGTTTGCAGTCGCGCCATGCATAAG
S106A forward	TTGGTGGGACATGCGCAAGGTGGCGTA
S106A reverse	TACGCCACCTTGCGCATGTCCCACCAA
D138A forward	CAATTAAAGGATGCGGCCTTAAATGGT
D138A reverse	ACCATTTAAGGCCGCATCCTTTAATTG
Q145A forward	AATGGTGACACTGCGGGCGCAACTTAT
Q145A reverse	ATAAGTTGCGCCCGCAGTGTCACCATT
I154A forward	AATCCTGAACACGCGCCAGCAGCTATT
I154A reverse	AATAGCTGCTGGCGCGTGTTCAGGATT

References

1. Lai K.K.; Lorca G.L.; Gonzalez C.F. Biochemical properties of two cinnamoyl esterases purified from a *Lactobacillus johnsonii* strain isolated from stool samples of diabetes-resistant rats. *Appl. Environ. Microbiol.* **2009**, *75*, 5018–5024. [doi: 10.1128/AEM.02837-08] [PubMed PMID: 19502437] [PubMed Central PMCID: PMC2725488].

2. Goldstone, D.C.; Villas-Bôas, S.G.; Till, M.; Kelly, W.J.; Attwood, G.T.; Arcus, V.L. Structural and Functional Characterization of a Promiscuous Feruloyl Esterase (Est1E) from the Rumen Bacterium *Butyrivibrio Proteoclasticus*. *Proteins Struct. Funct. Bioinforma.* **2010**, *78*, 1457–1469. [doi: 10.1002/prot.22662] [PubMed PMID: 20058325].

3. Aschauer, P.; Zimmermann, R.; Breinbauer, R.; Pavkov-Keller, T.; Oberer, M. The Crystal Structure of Monoacylglycerol Lipase from M. Tuberculosis Reveals the Basis for Specific Inhibition. *Sci. Rep.* **2018**, *8*, 8948. [doi: 10.1038/s41598-018-27051-7] [PubMed PMID: 29895832] [PubMed Central PMCID: PMC5997763].

4. Labar, G.; Bauvois, C.; Borel, F.; Ferrer, J.L.; Wouters, J.; Lambert, D.M. Crystal Structure of the Human Monoacylglycerol Lipase, a Key Actor in Endocannabinoid Signaling. *ChemBioChem* **2010**, *11*, 218–227. [doi: 10.1002/cbic.200900621] [PubMed PMID: 19957260].

5. Franklin, M.C.; Cheung, J.; Rudolph, M.J.; Burshteyn, F.; Cassidy, M.; Gary, E.; Hillerich, B.; Yao, Z.K.; Carlier, P.R.; Totrov, M.; et al. Structural Genomics for Drug Design against the Pathogen *Coxiella burnetii*. *Proteins Struct. Funct. Bioinform.* **2015**, *83*, 2124–2136. [doi: 10.1002/prot.24841] [PubMed PMID: 26033498].
